# Development and psychometric assessment of the sexual-reproductive health profile of women with type-1 diabetes mellitus

**DOI:** 10.1186/s12889-022-14400-5

**Published:** 2022-11-04

**Authors:** Nasrin Azimi, Giti Ozgoli, Abbas Ebadi, Hamid Alavi Majd, Assadollah Rajab, Zahra Kiani, Shahin Bazzazian, Tahereh Mokhtaryan-Gilani

**Affiliations:** 1 Present Address: Department of Midwifery, Karaj Branch, Islamic Azad University, Karaj, Iran; 2 Department of Midwifery and Reproductive Health, School of Nursing and Midwifery, Shahid Beheshti University of Medical Sciences, Tehran, Iran; 3 Behavioral Sciences Research Center, Life Style Institute, Baqiyatallah University of Medical Sciences, Tehran, Iran; 4 Faculty of Nursing, Baqiyatallah University of Medical Sciences, Tehran, Iran; 5 Department of Biostatistics, School of Allied Medical Sciences, Shahid Beheshti University of Medical Sciences, Tehran, Iran; 6 Iranian Diabetes Society, Tehran, Iran

**Keywords:** Sexual health, Reproductive health, Women, Psychometrics properties, Profile, Type-1 diabetes mellitus

## Abstract

**Background:**

To improve the sexual-reproductive health (SRH) of women with type-1 diabetes mellitus (T1DM), plans should be formulated on the basis of the existing situation. The situation can be examined by a valid tool conforming to the specific domains of SRH in the target group. The present study seeks to develop and assess the psychometric properties of the SRH Profile of Women with T1DM.

**Method:**

Based on the extracted concepts of SRH Profile of Women with T1DM in a previously conducted study, a tool was designed using the following steps: The selection of the conceptual model, explaining the objectives, designing the roadmap and development of the tool. In the psychometric assessment phase, the content, face and construct validity (convergent validity and principal component analysis) were assessed with the participation of 365 married women of reproductive age with T1DM. The reliability of the tool was determined by the internal consistency method (Cronbach’s alpha) and its stability using the test-retest method (Intraclass Correlation Coefficients).

**Results:**

The SRH Profile of Women with T1DM was formed with 53 items in two sections. Twenty-six items were about safe motherhood and reproductive system; 27 items were about the three components of concerns about the reproductive system health and function, sexual health and function, and violence related to T1DM. The three components in the second section explained 49.44% of the total variance. Cronbach’s alpha was 0.872 and the total intra-cluster correlation coefficient was 0.946.

**Conclusion:**

SRH Profile of Women with T1DM is a valid, reliable, and specific tool for assessing sexual-reproductive health in women with type-1 diabetes mellitus.

**Supplementary Information:**

The online version contains supplementary material available at 10.1186/s12889-022-14400-5.

## Introduction

Type-1 diabetes mellitus (T1DM) is a complex metabolic disorder characterized by chronic hyperglycemia following the destruction of pancreatic beta cells by the immune system and the complete lack of insulin [1]. According to the latest statistics by the International Diabetes Federation in 2019, 463 million adults in the world and 9.6% of the adult population in Iran have diabetes [2]. T1DM accounts for around 10% of all diabetes cases [3]. The prevalence of T1DM in the world, Asia, and Iran is 9.5, 6.9, and 12.1 cases per 10,000 people, respectively [4]. Of all the women with diabetes in Iran, 36% have T1DM [5].

In addition to the known effects of the disease on different parts of the body, long-term diabetes in women poses challenges in life, especially concerning sexual-reproductive health (SRH) [6]. Chronic hyperglycemia and complete lack of insulin throughout the patient’s lifespan exert toxic effects on the brain and ovaries and also damage the artery walls and autonomic nervous system and thus adversely affect the sexual and reproductive system function [7, 8]; They also cause late puberty, menstruation disorders, ovarian dysfunction, infertility, and premature menopause [9]. In women, sexual function is associated with changes in blood flow and increased risk of genital infections, reduced vaginal lubrication [10], and long-term complications such as neuropathy [11]. Pregnant women are more likely to carry excess weight and have hypertension, dysglycemia, preeclampsia, cesarean section, and infections. The infants of these mothers are prone to congenital anomalies, preterm birth, premature neonatal death, and macrosomia, followed by difficult delivery and damage to the brachial plexus [12]. People with diabetes are at higher risk for mental disorders and distress, which jeopardizes treatment adherence and increases the risk of serious disease-induced complications [13].

Given the adverse effects of T1DM on the SRH of women, the first step in promoting SRH is to learn the existing conditions using a valid tool that conforms to the specific domains of SRH in the target group.

SRH refers to complete social and psychophysical well-being in all dimensions associated with the reproductive system and sexuality [14]. The right of humans to make choices with respect to their SRH includes choosing a safe and satisfactory sexual life and making personal decisions about their reproduction [15]. To ensure their SRH, health authorities need to devise comprehensive methods and definitions [16].

SRH-promoting approaches consist mainly of offering sexual and reproductive counselling, training and services in areas including postnatal, birth and antenatal care, emergency obstetric and newborn care, preventing and treating sexually-transmitted diseases (STD) such as HIV, safe abortion and post-abortion care and regulations, referral and emergency services for victims of gender-based and sexual violence, preventing, diagnosing and managing reproductive cancers, particularly cervical cancer, and providing information, counselling and relevant services for infertility, subfertility and SRH [14].

The first step in the promotion of SRH among women with type-1 diabetes mellitus (T1DM) is to assess their SRH status by a validated tool. An extensive review of literature on the tools available for measuring SRH in T1DM patients revealed numerous tools; for instance:

The Depression Anxiety and Stress Scale (DAS) [17, 18], Diabetes Integration Scale (ATT-19), Diabetes-39 (D39) [19], Zung Self-Rating Depression Scale (ZUNG:DS) [20], Female Sexual Function Index (FSFI) [21], Beck Depression Inventory (BDI) [22], Audit of Diabetes-Dependent Quality of Life (ADDQOL) [23], Diabetes Treatment Satisfaction Questionnaire (DTSQ) [23], Hamilton Depression Rating Scale (HDRS) [24], Diabetes Health Profile (DHP) [25], 36-Item Short Form Survey (SF36) [26], Diabetes Self-Management Questionnaire (DSMQ) [27], Diabetes Quality Of Life (DQOL) [28], Hudson’s Index of Sexual Satisfaction [29], Reproductive Health Attitudes and Behavior (RHAB) [30], Swedish Diabetes Empowerment Scale (SWE-DES-23) [31], Derogatis Sexual Functioning Inventory (DSFI) [32], Female Sexual Distress Scale (FSDS) [32], Golombok Rust Inventory of Sexual Satisfaction (GRISS) [33].

The concepts assessed in these questionnaires are not comprehensive for measuring the domains of SRH in people with T1DM, and some of the questionnaires are too long and often revolve around concepts such as diabetes quality of life [34], general health [26], empowerment of people with type-1 and type-2 diabetes [31], patients’ care behaviors [27, 28], diabetes control [34], satisfaction with disease control [28], blood sugar control [27, 34], satisfaction with treatment [23], social life and diabetes [23, 27, 34], daily activities and exercise in diabetes [23, 27, 34], barriers to activity [25], nutrition [23], food restrictions [25], women’s sexual function [21], marital satisfaction [27, 33], sexual distress [35], diabetes concerns [25, 34], mental adaptation and attitude toward diabetes [18], depression [17, 20, 22, 24], anxiety [17], stress [17], sexual-reproductive health of adolescent girls with T1DM, and preconception counseling and measures [36]. Some of the tools assessing these concepts are non-specific and do not include all the components of sexual-reproductive health in T1DM women.

The New Dimension Consulting (NEDICO) and United Nations Population Fund (UNFPA) have developed a questionnaire with 114 items to measure women’s sexual-reproductive health [37]. Their questionnaire is not designed for people with chronic diseases (such as diabetes mellitus); therefore, its items do not take account of specific healthcare considerations or the negative consequences and complications of T1DM for the reproductive system. RHAB, on the other hand, was designed to address reproductive health-related behavior in T1DM and is concerned with adolescents and includes only concepts related to achieving normal blood sugar and getting preconception and contraception counseling. RHAB is thus not comprehensive for use among both adolescents and women of reproductive age [30]. Given the observed lack of specific tools with items corresponding to the concept and dimensions of SRH affected by T1DM, the present study was conducted to develop and assess the psychometric properties of the Sexual-Reproductive Health Profile of Women with Type-1 Diabetes Mellitus (SRHP of WT1DM).

## Methods

### Study design

This paper is reporting a part of a larger scale exploratory, sequential, mixed-methods study that was performed in two phases, including development of a framework to study sexual and reproductive health of women with type-1 diabetes mellitus (that is reported elsewhere [37, 38]) and psychometric assessment of a drafted preliminary tool for application in assessment of the sexual-reproductive health profile of these women.

### Tool design and item generation

The design of the tool and the generation of the item pool were based on the four-step model of Waltz et al. [39], including: 1- The selection of the conceptual model; 2- Explaining the objectives; 3- Designing the roadmap; and 4- Development of the tool.

A panel of experts was formed with the presence of sexual health experts, endocrinologists, pediatricians (the main representatives of diabetes education in Iran, who have been closely involved in the education and treatment of patients with T1DM for many years), and gynecologists to complete and review the results, and their comments were reviewed and applied.

### Step one

The conceptual model was selected from the previously conducted study [38].

### Step two

The objectives were defined based on the modified domains of women’s SRH [38].

### Step three

The initial estimation of the items was performed to design the roadmap/blue-print [37, 38].

### Step four

To produce the pool of items, concepts developed in [38] were used. The generated items underwent psychometric assessment. The qualitative content validity and qualitative face validity of the items were also examined based on [40].

### Psychometric assessment of the tool and item reduction

#### Face validity

The face validity of the study tool was assessed with the participation of ten women with T1DM using face-to-face interviews. Their comments were collected on the apparent appropriateness, comprehensibility, legibility, difficulty level, and ease of use of the items. These interviews also helped with the semantic convergence of the items with the patients’ experiences. The necessary changes were made based on the comments to simplify the items.

#### Content validity

The content validity of this study was evaluated qualitatively and quantitatively based on [41]. In the qualitative step, 20 specialists, including obstetricians and reproductive health experts (*n* = 12), gynecology surgeons (*n* = 3), a pediatric endocrinologist (*n* = 1), and endocrinologists and internists (*n* = 4), checked the items in terms of grammar, use of appropriate words, and proper sequencing and placement of the items. Modifications were applied to the questionnaire according to their comments.

Quantitative content validity was also examined by calculating the content validity ratio (CVR) and content validity index (CVI) in two separate stages. In the CVR assessment stage, the experts examined the 57-item questionnaire in terms of the necessity of the items. According to Lawshe et al., the acceptable mean score with the participation of 20 experts is 0.42 and above [42]. After applying the necessary changes according to the comments, the questionnaire was also examined in terms of the CVI from the perspective of 20 other experts; that is, the relevance of the items to the research objectives was evaluated. The item-level content validity index (I-CVI), Kappa statistic and the scale-level content validity index average (S-CVI/Ave) were calculated as well based on [43].

### Pilot study: item analysis

The loop method was used to analyze the items [44], and the correlation between the items (acceptable limit: 0.7 and above), the corrected correlation coefficient between the items and the total score (acceptable limit: 0.2 and above), and the effect of removing each item on Cronbach’s alpha changes were assessed. For this purpose, a pilot study was conducted on 30 eligible women with T1DM.

When estimating the sample size of a pilot trial, the simplest method is to apply sample size rules of thumb [19]. A flat rule of thumb is a single number that is suggested for every situation. For example, ‘30’ is a popular number [20].

The decision to retain or remove the items was made based on the correction coefficient and the effect of item removal on Cronbach’s alpha coefficient.

### Construct validity

The principal component analysis (PCA) and convergent validity were used to evaluate the construct validity of the items. Twenty-six of the items included concepts related to the effects of T1DM on safe motherhood and the reproductive system. Based on the item themes, the designed responses took the form of a nominal scale that differed from the subsequent items’ responses; therefore, these questions, which had a different structure, were placed as a separate part of the tool and the remaining 30 items were analyzed by PCA. To assess the construct validity, a cross-sectional study with purposive sampling was performed in the Iranian Diabetes Association from June 2020 to January 2021.

Married women willing to participate in the study with at least 1 year since their T1DM diagnosis were invited by telephone calls to join the study. As a general rule, three to ten samples per item appears sufficient for verifying construct validity [45]. A total of 365 people were recruited to this study.

The analysis was performed using the principal component analysis and Promax rotation. The minimum factor loading required to maintain the item was 0.4, and eigenvalues ​​​​had to be greater than 1.

### Convergent validity

Short Form-8 (SF-8) health survey or the short-form health-related quality of life instrument can be used to assess the health of people with chronic diseases and were utilized in this study to assess the convergent validity [46].

To establish convergent validity, strong moderate correlations (0.5–0.7) are often preferred. If the correlation between the score of the new test and the previous test is very high, the new test does not have much new information compared with the previous one [47]. A correlation value of 0.5 was considered acceptable in this study.

### Reliability

Internal consistency (based on Cronbach’s alpha coefficient) and stability were measured using the test-retest method (based on ICC) to determine reliability. Cronbach’s alpha coefficient was calculated for the components and also the entire tool to evaluate the internal consistency of the instrument [39]. To assess stability, the questionnaire was completed by 30 women with T1DM twice at 2 weeks’ interval, and the ICC was calculated for the components and the entire scale as well. The minimum acceptable Cronbach’s alpha coefficient and ICC were 0.7 in this study.

### Responsiveness and interpretability

According to the Consensus-based Standards for the Selection of Health Status Measurement Instruments (COSMIN) checklist, which is a comprehensive measure of the quality of health instruments, in addition to validity and reliability, responsiveness and interpretability are also important criteria in instrument psychometrics [48].

### The criteria for interpretability and the optimal values were

- Determining the percentage of unanswered items; optimal value = 10–20% [49]
- Sampling adequacy; optimal limit ≥0.8 [50]
- Determining ceiling and floor effects, optimal value < 20% [51]

### The criteria for responsiveness and the optimal values were

- Standard error of measurement (SEM)
  SEM = SD _Pooled_ √1– ICC [40].
- Smallest Detectable Changes (SDC) or Minimum Detectable Changes (MDC)
  MDC (SDC) = SEM X 1.96 X √2, MDC% = (MDC / mean) 100.

A MDC percentage **<** 30% is acceptable and values less than 10% are considered excellent [52].

### Scoring

The scoring of the items in the safe motherhood component was such that a better condition got a higher score and the reverse. Therefore, 26 items were scored from 1 to 3 and the rest of the items were scored on a 5-point Likert scale from 1 to 5; four items were scored inversely, in which a higher score was considered more desirable.

If possible, it is recommended to use summative scoring procedures to calculate the total score or subscale score of a tool. As such, a higher score indicates better conditions and a lower score poorer conditions [39]. Standardization to 100 was applied to better understand the scoring and compare the scores of the different subscales [53].

$$\textrm{Score}\ \textrm{in}\ \textrm{percent}=\frac{\textrm{The}\ \textrm{raw}\ \textrm{score}\ \textrm{obtained}-\textrm{Minimum}\ \textrm{possible}\ \textrm{score}}{\textrm{Maximum}\ \textrm{possible}\ \textrm{score}-\textrm{Minimum}\ \textrm{possible}\ \textrm{score}} \times 100$$

### Ethical considerations

This study was approved by the Ethics Committee of Shahid Beheshti University of Medical Sciences (IR.SBMU.PHARMACY.REC.1399.218). Participation in the study was fully voluntary and contingent on consent. To maintain the confidentiality of participants’ data, the questionnaires were completed anonymously with no identification number.

## Results

### Item generation

According to selecting the articles related to the research objectives to generate the pool of items [38], about the terms AIDS and T1DM, a search in literature showed that articles on this subject mostly deal with the effects of anti-HIV medications on blood sugar and insulin and the risk factors of diabetes (i.e., they do not assess AIDS in women with T1DM). Therefore, the appropriate items measuring this dimension were placed in the pool of items based on the panel of experts’ opinion and the NEDICO questionnaire [54].

In studies on mental health (as part of the concept of sexual reproductive health) among patients with type-1 diabetes, the most common tools dealt with public mental health, and their emphasis was anxiety and depression. Based on the recommendations of the panel of experts, the research team relied on the components derived from the concept of SRH in T1DM [38] to design items covering the concerns about reproductive system health and function in connection with type-1 diabetes and the items were added to the pool of items.

The tool development of this study resulted in the total number of 120 generated items. The similar items were removed, decreasing the remaining items to 96. According to the research team, the items with similar concepts were also merged, and the pool of items reaching the validity phase was 57.

### Psychometric assessment stage

#### Face validity

Based on participants’ suggestions, explanations were provided between parentheses for words such as ‘orgasm’ and ‘arousal’ to clarify the meaning of the item, and some verbs and items were simplified.

#### Content validity

The recommendations of the panel of experts about the content of the items were applied for qualitative content validity assessment. In the CVR assessment, one item was removed due to a CVR less than 0.42. In the CVI assessment, the I-CVR for all the items was in the range of 0.85–1, the modified Kappa was in the range of 0.84–1, and the total S-CVI/Ave was 0.96.

### Item analysis

This tool has two parts. The first part has 26 items on safe motherhood and the reproductive system. The variables assessed in these items were related to themes such as the status of the reproductive system functions and specific measures and considerations for pre-pregnancy, during pregnancy, during childbirth, and postpartum in T1DM patients, maternal, fetal, and neonatal consequences in T1DM patients, family planning, and genital infections, which had three-choice answers. The correction coefficient for these items, which differed from the other 30 items both in their nature and response scale, was less than 0.2 (− 0.42–0.11).

The second part of the questionnaire contained 30 items related to concerns about the reproductive system health and functions, sexual health and function, and violence related to T1DM. In this part, Cronbach’s alpha of each item was above 0.7 (α = 0.86–0.88) and the total Cronbach’s alpha coefficient was 0.88, and the omission of none of the 30 items had additional effects on the total Cronbach alpha value; therefore, all the items were retained.

### Construct validity

At this stage, for a cross-sectional study and to check the construct validity, the 56-item tool was sent to 365 eligible participants.

#### Demographic information

Table 1 presents the demographic characteristics of the women with T1DM.

**Table 1:** The demographic characteristics of the women with T1DM

Demographic Characteristics	Result
	Mean (SD)/ N (%)
Age (years)	35.68 (5.07)
Education (years)	14.56 (3.28)
Duration of T1DM (years)	16.69 (7.43)
Duration of marriage (years)	12.56 (6.98)
Husband’s age (years)	40.21 (6.95)
Husband’s education (years)	14.15 (3.65)
Employment rate (percent)	125 (34.24)
Family history of T1DM (percent)	104 (28.50)
Development of diabetes before marriage (percent)	225 (61.64)
Diagnosis with diseases other than T1DM (percent)	223 (61.09)
History of use of medications other than insulin (percent)	225 (61.64)
History of type-1 diabetes in the husband (percent)	6 (1.64)
History of type-2 diabetes in the husband (percent)	16 (4.38)

Three out of 30 items were excluded due to a factor loading less than 0.4, and the number of final items remaining in this part of the tool reached 27 after the PCA. The Kaiser-Meyer-Olkin (KMO) measure of sampling adequacy was 0.84 and Bartlett’s test of sphericity was χ^2^ = 4359.08 and *P* < 0.001. In this section, three components were obtained, which explained 49.44% of the total variance. Based on the meanings of the items, these components were named ‘concerns about the reproductive system health and functions’, ‘sexual health and function’, and ‘violence related to T1DM’. The eigenvalues ​​for these three components were 6.003, 4.996, and 2.366, respectively (Table 2).

**Table 2:** The results of the principal component analysis of the SRHP of WT1DM

Component	Item	Factor Loading	Communality	Variance Percentage	Eigenvalues
Concerns about the reproductive system health and functions	Are you worried about not having access to specialized and comprehensive counseling when choosing a safe method of contraception?	0.696	0.489	22.22	6.003
	Are you worried about the impact of diabetes on your ovarian function and health?	0.695	0.463		
	Are you worried about the worsening of diabetes following pregnancy?	0.691	0.536		
	Are you worried about the effect of diabetes on sexual function?	0.678	0.519		
	Are you worried about the potential side effects of contraceptives on diabetes (and diabetes control)?	0.655	0.444		
	Are you worried about possible changes in the amount and method of insulin intake during pregnancy?	0.651	0.489		
	Are you worried about an increased risk of genital infections caused by your illness?	0.635	0.432		
	Are you worried about premature menopause for yourself?	0.634	0.387		
	Are you worried about your ability to get pregnant?	0.628	0.398		
	Are you worried about receiving pre-pregnancy counseling and specialized services?	0.627	0.455		
	Given the effects of uncontrolled diabetes on the pregnant woman and fetus, are you worried about an unwanted pregnancy and its consequences?	0.593	0.348		
	Are you worried about the effect of diabetes on your menstrual status?	0.578	0.348		
	Are you worried about the transmission of diabetes to the fetus or the health of the fetus?	0.578	0.407		
	Are you worried about your husband’s sexual dissatisfaction?	0.480	0.367		
Sexual health and function	Do you feel there is excitement in your sexual relations with your husband?	0.759	0.634	18.48	4.996
	Over the last 4 weeks, how often have you experienced sexual arousal during sexual activities?	0.758	0.548		
	Over the last 4 weeks, how often have you reached orgasm during sexual activities?	0.758	0.558		
	Over the last 4 weeks, how often have you achieved vaginal lubrication during sexual activities?	0.754	0.567		
	Over the last 4 weeks, how often have you wanted to engage in sexual activities?	0.725	0.539		
	Do you feel that there are common interests and preferences in your sexual relations with your husband?	0.692	0.550		
	Do you ask your husband to pleasure you during sex?	0.619	0.380		
	How satisfied have you been with your sexual relations with your husband over the past 4 weeks?	0.671	0.508		
	Is intimacy an important part of sex for you and your husband?	0.528	0.331		
Violence related to T1DM	Have you ever been verbally abused by someone other than your husband (parents or acquaintances) because of your illness?	0.783	0.605	8.74	2.366
	Have you ever been beaten by someone other than your husband (parents or acquaintances) only because of your illness (and for no other reason)?	0.680	0.453		
	Have you ever been verbally abused by your husband because of your illness?	0.580	0.361		
	Have you ever been beaten by your husband only because of your illness (and for no other reason)?	0.550	0.307		

### Convergent validity

The correlation between the profile score and the SF8 score showed that *r* = 0.502 and *p* < 0.001; therefore, the convergent validity of the tool was approved.

### Reliability

In examining the internal consistency, Cronbach’s alpha coefficient for the items of the three component was α = 0.877, and the total alpha coefficient of the 53-item profile (considering the 26 items of safe motherhood and reproductive system in the first part of the profile) was α = 0.872. Internal stability was also assessed by the test-retest method. Table 3 presents the ICC of the components as well as the entire profile, also the ICC of safe motherhood and reproductive system section 0.953, 95% CI = 0.823–0.956 and Cronbach’s alpha 0.583, *P* < 0.001. Table 4 also presents the mean raw scores and the 0–100 scoring of the participants in the study. Participants’ mean (SD) raw scores and 0–100 scoring for the safe motherhood and reproductive system section were 67.17 (4.30) and 79.17 (8.27) respectively.

**Table 3:** Intra-class correlation coefficients of the SRHP of WT1DM by dimension and overall

Component	Cronbach’s alpha	ICC	95% CI	***P***-value
Concerns about the reproductive system health and functions	0.88	0.866	0.717,0.936	*p* < 0.001
Sexual health and function	0.84	0.966	0.928,0.984	*p* < 0.001
Violence related to T1DM	0.63	0.919	0.831,0.961	*p* < 0.001
concerns about the reproductive system health and functions, sexual health and function, violence related to T1DM	0.877	0.937	0.867,0.970	*p* < 0.001
Total (SRHP of WT1DM-53 item)	0.872	0.946	0.887,0.974	*p* < 0.001

**Table 4:** Participants’ mean raw scores and 0–100 scoring for the SRHP of WT1DM

Component	Number of Items	Mean (SD) (Raw Score)	Mean (SD) (0–100)
Concerns about the reproductive system health and function	14	47.33 (12.30)	59.53 (21.97)
Sexual health and function	9	32.48 (7.32)	66.23 (20.35)
Violence related to T1DM	4	19.19 (1.69)	94.94 (10.56)
Total (SRHP of WT1DM)	53	166.54 (18.43)	74.97 (10.24)

### Responsiveness and interpretability

Table 5 shows the results of assessing responsiveness and interpretability. The ceiling and floor effect of safe motherhood and reproductive system section were both zero, also the %MDC and SEM in this section was 3.82 and 0.93.

**Table 5:** Responsiveness and interpretability of the SRHP of WT1DM

Interpretability		Responsiveness	
Percentage of unanswered items	Ceiling and floor effect	MDC%	SEM
0%	Concerns about the reproductive system health and function: 1.1/0 Sexual health and function: 1.6/0.3 Violence related to T1DM: 68.80/0	First component: 24.74 Second component: 11.48 Third component: 8.02	First component: 4.24 Second component: 1.35 Third component: 0.56

According to this table, the interpretability and responsiveness of the profile are confirmed.

Regarding the ceiling effect of the violence components affected by type 1 diabetes, based on the profile scoring system, in which higher scores denote better health, when participants score higher, they have been subject to less violence (reverse scoring). Therefore, the ceiling effect of 68.8% does not denote a negative score; rather, it indicates a better situation.

## Discussion

Based on investigations, to date, no valid tools have been designed and validated to measure the concept and domains of the SRHP of WT1DM. This study was conducted to design and evaluate the psychometric properties of the SRHP of WT1DM based on an exploratory, sequential, mixed-methods study. The resultant profile had good validity and reliability. The SRHP of WT1DM consists of two parts. The first part has 26 items about the components of safe motherhood and reproductive system in T1DM, and the second part has 27 items in the three components of concerns about the reproductive system health and functions (14 items), sexual health and function (nine items), and violence related to T1DM (four items), which explained 49.44% of the total variance, with the share of each component being 22.22, 18.48 and 8.74% of the variance, respectively.

In the present study, the SRHP of WT1DM included items related to the components of safe motherhood and reproductive system and the three components of concerns about the reproductive system health and functions, sexual health and function, and violence related to T1DM. Stenhouse et al. [6] discussed the most important effects of type-1 and type-2 diabetes on the female reproductive system in menarche, menstrual disorders, contraception, sexual dysfunction, pregnancy, and menopause. Although there are similarities between the present study and Stenhouse’s study in terms of the main issues of reproductive health, the present study also thoroughly explored themes such as postpartum outcomes, specific considerations in preconception programs, and pregnancy in T1DM and provides a more comprehensive view of the domains of women’s sexual-reproductive health. Validation steps have also been taken for this tool and it can be used as a valid profile for measuring the sexual-reproductive health status of women with T1DM.

Women’s Sexual Reproductive Health Questionnaire by NEDICO, which has 114 items and six components and was used for determining the components of the questionnaire in the present study [54], is suitable for measuring the SRH of women in a healthy population and therefore does not include specific questions about sexual-reproductive health affected by T1DM and disease-specific considerations.

The first part of the SRHP of WT1DM examines the components of safe motherhood and reproductive system in T1DM. It has 26 appropriate items on health status and reproductive system function covering areas such as puberty, menstruation, fertility, and menopause, measures and considerations before and during pregnancy and childbirth, maternal and fetal outcomes in pregnancy and childbirth, neonatal outcomes, postpartum measures, considerations, and consequences, and considerations in family planning. The items have been extracted from a comprehensive review of relevant literature and are designed in congruence with the components of safe motherhood and reproductive system in T1DM; therefore, they can cover the components and specific considerations of the safe motherhood and reproductive system components in T1DM in a specific and comprehensive manner.

The RHAB questionnaire, which is based on interviews and health models with the participation of adolescent girls with T1DM with an average age of 17.9 years, has been designed to assess reproductive health behaviors and attitudes. Based on the results of the exploratory factor analysis, the questionnaire was developed with 24 items in the three components of achieving normal blood sugar, accessing preconception counseling, and contraception, which explained 55% of the total variance. The internal consistency of the questionnaire was α = 0.65.0.83 [30]. In the SRHP of WT1DM, preconception counseling and contraceptive methods were included in the safe motherhood category, and due to the nature of the items and the response options, which could not be formatted as a Likert scale, this section of the SRHP of WT1DM was not component analyzed, and its construct validity was assessed using the convergent validity method against the SF8. The internal consistency of the SRHP of WTD1M was α = 0.63–0.88, which is close to that of the RHAB questionnaire.

The second part of the profile, which is designed with 27 items in three components, examines the concerns of women with T1DM in reproductive ages about the effect of the disease on reproductive and sexual functions. Also, the violence dimension in the SRHP of WT1DM specifically examines the violence induced by the disease. Furthermore, the items related to the sexual dimension have an appropriate validity in the target group based on the psychometric assessment of the study group.

The sexual dimension of the SRHP of WT1DM has nine items. The items in this section of the profile were selected from the tools used in studies on T1DM for measuring sexual health and function, which were retrieved from the literature review. Furthermore, they were validated according to the comments of the expert panel and research team for married women with T1DM at reproductive age. The selected questions were chosen from tools such as FSFI, DAS, and GRISS. Cronbach’s alpha of this dimension was obtained as 0.84. A convergent validity assessment against the SF8 was also carried out in addition to the PCA to evaluate the construct validity of the tool, which yielded *r* = 0.502.

FSFI is one of the most important tools used in studies to measure sexual performance and health in women with T1DM [55] that entails 19 items in six components related to sexual response and function and its reliability is α = 0.82. FSDS is concerned with female sexual distress with one components and 12 items and has a reliability of α = 0.86 [35]. DAS has 32 items in four components and its convergent validity is *r* = 0.80 and internal consistency α = 0.96 [56]. GRISS deals with sexual satisfaction with 56 items in four components, and its validity has been approved by discriminant validity assessment, and its reliability is *r* > 0.7 using test-retest and the split-half method [33]. DSFI has 254 items in ten components and a reliability of α = 0.6–0.97 [32]. Each of the mentioned tools, cover some limited aspect of the SRH which is a multidimensional concept. To cover more aspects in a single tool the current tool was developed.

In the SRHP of WT1DM, the mental dimension focuses on concerns about the effect of T1DM on reproductive system health and functions and sexual function in women of reproductive age using 14 items. Cronbach’s alpha coefficient of this dimension is 0.88 and its intra-cluster coefficient is 0.86. The results of the study showed a weak correlation between the dimensions of the SRHP of WT1DM and SF8, which indicates that the two questionnaires evaluate different concepts. Valid yet general tools have been used in studies on mental health and T1DM [20, 22, 24, 25], but none of them have items compatible with the concerns of women with T1DM about the effect of the disease on their reproductive and sexual functions. The DHP is designed with 32 items in three components and has a reliability of *r* > 0.7. Fourteen of the DHP items examine psychological distress in diabetes, including bad temper, feeling of hopelessness, irritability, self-harm, and feeling hostility from others, and the profile lacks a statement about sexual and reproductive health-related distress.

In the reliability assessment stage, Cronbach’s alpha coefficient, overall intra-cluster correlation coefficient, and the components of the SRHP of WT1DM were optimal, indicating that this profile can be proposed as a valid and reliable tool.

### Limitations and strengths

While, we collected the opinions of the experts on the patients’ perspective in addition to their own opinion, the patients themselves were not present in the expert panel. Some of the viewpoints of the patients, such as the effect of psycho-social coercion might be missed. For example, the effect of social pressure of family members on abortion, or the pressure from the partner’s side on pregnancy while the patient is not ready herself. According to the concept development study [38] which was used to generate the pool of items, there is no comprehensive study regarding the concept of sexual and gender based violence (GBV) affected by T1DM. Thus, this concept is not assessed in the present tool. We suggest to conduct a hybrid study in which the combination of the viewpoints of the patients as well as literature review is used to develop concepts; these concepts can be used to modify the items of the current tool. The score (0–100) derived from the current tool was divided into quartiles to assist better comprehension of statistics. Further studies are needed to determine real cut-offs and classification for this score.

One of the strengths of the tool was its simplified items and the provision of additional explanations for some items to clarify the concept. Also, despite the wide range of search terms and phrases and the prolonged process of search in the databases, an in-depth and comprehensive examination of the concept and dimensions of SRH was achieved in the target group, and a valid, reliable, and specific tool was finally designed for the measurement of this concept that did not exist in previous studies.

## Conclusion

The SRHP of WT1DM includes 26 items in the safe motherhood and reproductive system dimension with three-choice answers and 27 items in the three dimensions of concerns about the reproductive system health and functions, sexual health and function, and violence related to T1DM.

The SRHP of WT1DM and the findings of the present study might be used in health care centers not only to assess the current situation, but also to develop programs to promote the SRH of women with T1DM.

The viewpoints of the patients themselves should be obtained through further studies and the current tool might be modified accordingly. Further studies are needed to determine classification and cut-offs according to the score derived from the current tool.

## Supplementary Information

**Additional file 1.**

## Data Availability

The datasets used and/or analyzed during the current study are available from the corresponding author on reasonable request.

## Abbreviations

T1DM: Type-1 Diabetes Mellitus
SRH: Sexual-Reproductive Health
SRHP of WT1DM: Sexual-Reproductive Health Profile of Women with Type-1 Diabetes Mellitus
WHO: World Health Organization
UNFPA: United Nations Population Fund
STD: Sexually Transmitted Diseases
GBV: Gender-Based Violence
DAS: Depression, Anxiety and Stress Scale
ATT-19: Diabetes Integration Scale
D-39: Diabetes-39
SRDS: ZUNG: DS: Zung Self-Rating Depression Scale;
FSFI: Female Sexual Function Index
BDI: Beck Depression Inventory
ADDQOL: Audit of Diabetes-Dependent Quality of Life
DTSQ: Diabetes Treatment Satisfaction Questionnaire
HDRS: Hamilton Depression Rating Scale
DHP: Diabetes Health Profile
SF-36: Short Form-36 Health Survey
DSMQ: Diabetes Self-Management Quality Questionnaire
DQOL: Diabetes Quality Of Life
RHAB: Reproductive Health Attitudes and Behavior
SWE-DES-23: Swedish Diabetes Empowerment Scale
DSFI: Derogatis Sexual Functioning Inventory
FSDS: Female Sexual Distress Scale
GRISS: Golombok Rust Inventory of Sexual Satisfaction
NEDICO: New Dimension Consulting
CVR: Content Validity Ratio
CVI: Content Validity Index
I-CVI: Item-level Content Validity Index
S-CVI/Ave: Scale-level Content Validity Index Average
PCA: Principal component analysis
ICC: Intra-class Correlation Coefficients
KMO: Kaiser-Meyer-Olkin
COSMIN: Consensus-based Standards for the Selection of Health Status Measurement Instruments
SEM: Standard Error of Measurement
SDC: Smallest Detectable Changes
MDC: Minimum Detectable Changes
