# Supplementary material for: Development and psychometric assessment of the sexual-reproductive health profile of women with type-1 diabetes mellitus
Source: BMC Public Health. 2022 Nov 4;22:2018. doi: 10.1186/s12889-022-14400-5 (PMC9636675; doi:10.1186/s12889-022-14400-5)
Supplement: Supplementary file 1 — Additional file 1. [file 12889_2022_14400_MOESM1_ESM.docx]

This file contains the Sexual-Reproductive Health Profile of Women with Type-1 Diabetes Mellitus (SRHP of WT1DM). This tool has 53 items organized in four sections: **Safe motherhood and reproductive system** (items 1 to 26); **Concerns about the reproductive system health and functions** (items 27 to 40); **Sexual health and function** (items 41 to 49), **Violence related to T1DM** (items 50 to 53). Sections are provided in a sperate tables, the name of the section is included in the table title. The answer options are provided for each question in the form of rank or Likert. The scores are mentioned in the table, the more favorable the situation, the higher the score.

**Sexual-Reproductive Health Profile of Women with Type-1 Diabetes Mellitus (SRHP of WT1DM):** **Safe motherhood and reproductive system in T1DM**

| **Question**  **number** | **Item** | **Answer** | **Score** |
| --- | --- | --- | --- |
| 1 | At what age did you notice signs of puberty in your body? (signs such as the growth of breast buds and rough hair in the genital area and axillary) | 1. Between 8 -15 years old 2. After 15 years old 3. Before 8 years old | 3  2  1 |
| 2 | How is your menstruation? | 1. Regular 2. Irregular 3. I do not menstruate | 3  2  1 |
| 3 | How is your ovary function? | 1. No problem 2. I have polycystic ovary syndrome (PCO) 3. I have premature menopause (before 45y) | 3  2  1 |
| 4 | How is your pap smear test? | 1. I do it regularly according to the advice of my doctor or health staff. 2. I do not it regularly according to the advice of my doctor or health staff. 3. I did not do this test | 3  2  1 |
| 5 | How do you check your breast health? | 1. I examine my breasts every month and have a mammogram or ultrasound if my doctor recommends it. 2. Every month I only examine my breasts or have a doctor's examination or sonography and mammography. 3. I do nothing to assess breast health | 3  2  1 |
| 6 | Do you have a history of infertility? | 1. No 2. I do not know 3. Yes | 3  2  1 |
| 7 | Did you receive Specialized counseling before deciding to become pregnant? (Specialized counseling for eye, heart, kidney, thyroid, endocrine, internal medicine, nutrition, obstetrics and gynecology) | 1. I did all the Specialized counseling requested. 2. I just did some of the Specialized counseling mentioned. 3. The doctor recommended the Specialized counseling, but I did not. | 3  2  1 |
| 8 | Did you follow the Specialized counseling and recommendation of pregnant mothers with diabetes during pregnancy? (such as Specialized counseling and recommendation; Changes in insulin intake or type Changes in the frequency of blood sugar control, changes in meals, consultation with endocrinologists, internal medicine, cardiology and ophthalmology ( | 1. I followed the doctor's Specialized counseling and instructions. 2. Despite the doctor's advice, I only did some Specialized counseling and instructions. 3. Specialized counseling and instructions were requested by the doctor, but I did not do so | 3  2  1 |

**Sexual-Reproductive Health Profile of Women with Type-1 Diabetes Mellitus (SRHP of WT1DM):** **Safe motherhood and reproductive system in T1DM**

| **Question**  **number** | **Item** | **Answer** | **Score** |
| --- | --- | --- | --- |
| 9 | Did you have the ultrasound recommended by your doctor during your pregnancy? (NT assessment at 18-22 weeks, fetal abnormalities / 28th week, Doppler to evaluate placental function and fetal growth rate) | 1. I did all the ultrasounds requested by the doctor 2. I did some of the ultrasounds that the doctor requested 3. Despite my doctor's request and advice, I did not do any of the ultrasounds. | 3  2  1 |
| 10 | Did you get the following vaccines before (or during) pregnancy? (Influenza vaccine, pneumococcal vaccine, tetanus vaccine) | 1. Based on my vaccination history and doctor's recommendation, I injected all of these vaccines (I did not need to be vaccinated according to the instructions). 2. Despite my doctor's advice based on my vaccination history, I only injected some of the vaccines listed. 3. I did not receive any vaccine | 3  2  1 |
| 11 | How did you receive your prenatal care during your last pregnancy? | 1. I received regular based on the advice of my doctor and health team. 2. I received irregular 3. I did not receive any care. | 3  2  1 |
| 12 | How was your blood sugar status during your last pregnancy? | 1. I followed the medical recommendation and instructions for regulating blood sugar completely. 2. I followed some medical tips and recommendation to regulate blood sugar. 3. I did not follow medical recommendation and instructions. | 3  2  1 |
| 13 | During your last pregnancy, did these problems develop for the fetus? (Abnormalities, underweight, overweight, intrauterine growth retardation, oligo and polyhydramnios, abortion and death) | 1. There was no problem 2. Some of these problems occurred and I received the necessary medical care. 3. Some of these problems occurred and I did not receive the necessary medical care. | 3  2  1 |
| 14 | How was your last delivery? | 1. Normal vaginal delivery 2. Scheduled cesarean section 3. Emergency cesarean section | 3  2  1 |
| 15 | Did you have any of the following problems during your last pregnancy (for delivery)? (Severe hypoglycemia, hyperglycemia, hypertension, Decreased or impaired consciousness, bleeding and seizures) | 1. No particular problem occurred 2. Some of the mentioned problems were created which were controlled. 3. Some of the mentioned cases were created which were not well controlled and caused complications | 3  2  1 |
| 16 | What were the complications or problems in your infant during and after your last delivery? (Infant shoulder dystocia, fracture, the Shoulder Injuries, brachial palsy, seizures, respiratory problems, severe hypoglycemia, jaundice, breastfeeding problems, abnormalities, death) | 1. There was no complication. 2. A complication occurred but the infant was not admitted to the neonatal intensive care unit. 3. There was a complication and my infant was admitted to the neonatal intensive care unit. | 3  2  1 |

**Sexual-Reproductive Health Profile of Women with Type-1 Diabetes Mellitus (SRHP of WT1DM):** **Safe motherhood and reproductive system in T1DM**

| **Question**  **number** | **Item** | **Answer** | **Score** |
| --- | --- | --- | --- |
| 17 | Did you have any of the following problems after delivery? (Abnormal bleeding, seizures, infection, opening of cesarean section or episiotomy , vision problems, kidney problems, thyroid problems, Impaired blood sugar control status and HbA1c) | 1. No problem occurred. 2. Some problems occurred that were controlled by receiving medical care. 3. Some problems occurred that were not well controlled after receiving medical care and left complications. | 3  2  1 |
| 18 | How was your infant fed in the first 6 months? | 1. Exclusive breastfeeding 2. Exclusive breastfeeding and baby formula 3. Baby formula | 3  2  1 |
| 19 | How do you choose and change the method of contraception? | 1. We choose or change the contraception method based on the advice of the medical team and with the opinion of myself and my husband. 2. Without receiving advice from the medical team, we choose the method ourselves and change it with our own opinion. 3. Although I do not want to get pregnant, I do not have a method of contraception | 3  2  1 |
| 20 | What have been the side effects of using contraception for you? (Hyperglycemia, hypoglycemia, hyperinsulinemia, hyperlipidemia, hypertension, Coagulopathy, thrombosis, menstrual irregularities and spotting) | 1. I had no problem. 2. I had some side effects that were controlled by receiving counseling and care. 3. I had some complications that were not controlled and I had to change the method. | 3  2  1 |
| 21 | How do you get information about contraception? | 1. Doctor and health team 2. Media, Internet resources and books 3. Relatives and friends | 3  2  1 |
| 22 | Have you ever had an unplanned pregnancy? | 1. No 2. My pregnancy time was unintended pregnancy (I wanted to get pregnant at another convenient time) 3. My pregnancy was unwanted (I did not want to have children at all) | 3  2  1 |
| 23 | If you have an infection or disease in your genitals, how do you treat it? | 1. I had no problem. 2. I go to the doctor and take the necessary measures. 3. I do not go to the doctor and I treat myself. | 3  2  1 |
| 24 | How is your high-risk behavior? (Using a shared syringe and needles used by others is a common tool for tattooing and having sex outside of your marriage or spouse.) | 1. I do not have any high-risk behavior 2. I have some high-risk behaviors, but I have done the necessary tests to check for infection and disease. 3. I have some high-risk behaviors, but I have not done the necessary tests to check for infection and disease. | 3  2  1 |
| 25 | Has your doctor ever ordered an HIV test for you? (From the onset of diabetes) | 1. No 2. Yes, and I did. 3. Yes, and I did not. | 3  2  1 |
| 26 | Has your doctor ordered an HIV test in the last 12 months? | 1. No 2. Yes, and I did. 3. Yes, and I did not. | 3  2  1 |

**Sexual-Reproductive Health Profile of Women with Type-1 Diabetes Mellitus (SRHP of WT1DM):** **Concerns about the reproductive system health and functions**

| **Question**  **number** | **Item** | **Answer** | | | | |
| --- | --- | --- | --- | --- | --- | --- |
|  |  | **Always** | **Most of the time** | **Sometimes** | **Rarely** | **Never** |
|  |  | **Score** | | | | |
|  |  | **(1)** | **(2)** | **(3)** | **(4)** | **(5)** |
| 27 | Are you worried about the effect of diabetes on your menstrual status? |  |  |  |  |  |
| 28 | Are you worried about the impact of diabetes on your ovarian function and health? |  |  |  |  |  |
| 29 | Are you worried about premature menopause for yourself? |  |  |  |  |  |
| 30 | Are you worried about your ability to get pregnant? |  |  |  |  |  |
| 31 | Are you worried about receiving pre-pregnancy counseling and specialized services? |  |  |  |  |  |
| 32 | Are you worried about the worsening of diabetes following pregnancy? |  |  |  |  |  |
| 33 | Are you worried about possible changes in the amount and method of insulin intake during pregnancy? |  |  |  |  |  |
| 34 | Are you worried about the transmission of diabetes to the fetus or the health of the fetus? |  |  |  |  |  |
| 35 | Are you worried about not having access to specialized and comprehensive counseling when choosing a safe method of contraception? |  |  |  |  |  |
| 36 | Given the effects of uncontrolled diabetes on the pregnant woman and fetus, are you worried about an unwanted pregnancy and its consequences? |  |  |  |  |  |
| 37 | Are you worried about the potential side effects of contraceptives on diabetes (and diabetes control)? |  |  |  |  |  |
| 38 | Are you worried about an increased risk of genital infections caused by your illness? |  |  |  |  |  |
| 39 | Are you worried about the effect of diabetes on sexual function? |  |  |  |  |  |
| 40 | Are you worried about your husband's sexual dissatisfaction? |  |  |  |  |  |

**Sexual-Reproductive Health Profile of Women with Type-1 Diabetes Mellitus (SRHP of WT1DM): Sexual health and function**

| **Question**  **number** | **Item** | **Answer** | | | | |
| --- | --- | --- | --- | --- | --- | --- |
|  |  | **Always** | **Most of the time** | **sometimes** | **Rarely** | **never** |
|  |  | **Score** | | | | |
|  |  | **(5)** | **(4)** | **(3)** | **(2)** | **(1)** |
| 41 | Over the last four weeks, how often have you wanted to engage in sexual activities? |  |  |  |  |  |
| 42 | Do you ask your husband to pleasure you during sex? |  |  |  |  |  |
| 43 | Do you feel that there are common interests and preferences in your sexual relations with your husband? |  |  |  |  |  |
| 44 | Do you feel there is excitement in your sexual relations with your husband? |  |  |  |  |  |
| 45 | Is intimacy an important part of sex for you and your husband? |  |  |  |  |  |
| 46 | Over the last four weeks, how often have you experienced sexual arousal during sexual activities? | **Answer** | | | | |
|  |  | **I did not have sex** | **Always** | **Most of the time** | **Sometimes** | **Never** |
|  |  | **Score** | | | | |
|  |  | **(3)** | **(5)** | **(4)** | **(2)** | **(1)** |
|  |  |  |  |  |  |  |
| 47 | Over the last four weeks, how often have you reached vaginal lubrication during sexual activities? |  |  |  |  |  |
| 48 | Over the last four weeks, how often have you reached orgasm during sexual activities? |  |  |  |  |  |
| 49 | How satisfied have you been with your sexual relations with your husband over the past four weeks? | **Answer** | | | | |
|  |  | **I was very satisfied**  **dissatisfied** | **I was somewhat satisfied** | **Almost equal neither satisfied nor dissatisfied** | **I was somewhat satisfied** | **I was very dissatisfied** |
|  |  | **Score** | | | | |
|  |  | **(5)** | **(4)** | **(3)** | **(2)** | **(1)** |
|  |  |  |  |  |  |  |

**Sexual-Reproductive Health Profile of Women with Type-1 Diabetes Mellitus (SRHP of WT1DM): Violence related to T1DM**

| **Question number** | **Item** | **Answer** | | | | |
| --- | --- | --- | --- | --- | --- | --- |
|  |  | **Always** | **Most of the time** | **Sometimes** | **Rarely** | **Never** |
|  |  | **Score** | | | | |
|  |  | **(1)** | **(2)** | **(3)** | **(4)** | **(5)** |
| 50 | Have you ever been verbally abused by your husband because of your illness? |  |  |  |  |  |
| 51 | Have you ever been verbally abused by someone other than your husband (parents or acquaintances) because of your illness? |  |  |  |  |  |
| 52 | Have you ever been beaten by your husband only because of your illness (and for no other reason)? |  |  |  |  |  |
| 53 | Have you ever been beaten by someone other than your husband (parents or acquaintances) only because of your illness (and for no other reason)? |  |  |  |  |  |
